# Caregivers consequences of care among patients with eating disorders, depression or schizophrenia

**DOI:** 10.1186/s12888-015-0507-9

**Published:** 2015-06-09

**Authors:** Josune Martín, Angel Padierna, Bob van Wijngaarden, Urko Aguirre, Ane Anton, Pedro Muñoz, José M. Quintana

**Affiliations:** Research Unit, Galdakao-Usansolo Hospital, Barrio Labeaga s/n, Galdakao, 48960, Bizkaia Spain; Department of Psychiatry, Galdakao-Usansolo Hospital, Barrio Labeaga s/n, Galdakao, 48960 Bizkaia Spain; Health Services Research on Chronic Diseases Network - REDISSEC, Galdakao, 48960 Bizkaia Spain; Netherlands Institute of Mental Health and Addiction, Utrecht, The Netherlands; Department of Psychiatry, Ortuella Mental Health Center, Avenida del Minero n 1, Ortuella, 48530 Bizkaia Spain

**Keywords:** Caregivers, Eating disorders, Schizophrenia, Depressive disorders, Burden

## Abstract

**Background:**

The consequences of caring for a person with a mental illness can impose a substantial burden. Few studies have compared this burden among caregivers of patients with eating disorders and other mental illnesses. The objective of this study was to compare caregiver consequences in eating disorders (ED) with caregiver consequences in depression and schizophrenia, assessed with the same instrument, the Involvement Evaluation Questionnaire (IEQ). Another aim was to identify factors that may predict these consequences.

**Methods:**

We conducted a cross-sectional study involving 251 caregivers of ED patients; 252 caregivers of patients with depression; and 151 caregivers of patients with schizophrenia. Caregivers completed the Involvement Evaluation Questionnaire EU Version (IEQ-EU). Descriptive statistics, ANOVA, and Chi-square were applied to examine the inter-variable relationships. Consequences- indexes were also computed.

**Results:**

In all samples, worrying was the most commonly reported consequence of caregiving. Predictive variables for a high level of caregiver burden included being a mother or partner of the person being cared for (p = <.01), and being a caregiver of a patient with ED.

**Conclusions:**

The burden of caregiving is higher among caregivers of patients with eating disorders patients than among caregivers of patients with depression or schizophrenia. Our findings suggest that caregivers of patients with an ED could benefit from providing adequate assessment and support.

## Background

Mental illness in a close relative can be stressful for family members or friends, particularly those who are also the patient’s caregiver [1–4]. Such stress can lead to caregiver burden, which refers to problems, difficulties, or adverse events that affect the life of a patient’s significant other [5]. Several studies have evaluated the impact on burden of caregiving among individuals caring for patients with chronic disorders [6] such as schizophrenia [7–10], depression [11, 12], and eating disorders (ED) [13–16].

In recent years, there has been a growing concern for the consequences experienced by patient’s caregivers. One study using the Involvement Evaluation Questionnaire-EU Version (IEQ-EU) showed that the consequences of caring for an individual with depression or schizophrenia were comparable [11]. Other studies on the consequences of providing care for patients with affective disorders also suggest that the relatives of these patients experience considerable distress, sometimes strikingly similar to those in schizophrenia [17–19]. Depression affects daily routines and role functioning, poses a stress on interpersonal relations, and leads to symptoms of distress in spouses and children [11].

Caregivers of individuals with eating disorders must often struggle with their charges’ unwillingness to accept their illness, the outward signs of their malnutrition and the resulting social stigmatization, the daily struggles at meal times, and the frequent behavioral and mood alterations that often occur with ED [20]. Eating disorders can significantly affect family relationships and impose a substantial burden for caregivers. The consequences of providing care to an individual with an ED have been evaluated [13–15, 21, 22] and the results of these investigations suggest that caregivers have high levels of needs that are not usually addressed in clinical practice. Indeed, some authors have found that family caregivers of ED patients have higher levels of anxiety, depression, and perceived caregiving burden compared to caregivers of patients with other psychiatric illnesses [23, 24]. To our knowledge, these are the only two studies comparing psychological distress of caregivers of patients with ED or schizophrenia. The sample size of both pilot studies however was small (e.g., 30 caregivers of patients with schizophrenia and 32 of ED patients in the study of Graap et al., 2008b), and the results must be regarded as preliminary, as the authors indicated. Identifying factors that may predict caregiver burden among parental caregivers of ED patients could improve integrated health care strategies for this type of illness (Table 1).

**Table 1:** Consequences of caregiving across the three mental illness samples compared: eating disorders, depression, and schizophrenia

Caregivers of patients with				
	Eating Disorders^a^	Depression^b^	Schizophrenia^a^	
	(*n*=251)	(*n*=252)	(*n*=151)	
	Mean C-index*	Mean C-index*	Mean C-index*	***P*** **-value**
**IE Q-EU scales**				
Tension	0.18 (0.19)^b,c^	0.17 (0.21)^c,a^ ^b,a^ ^c,a^	0.11 (0.17)^b,a^ ^b,c^	**0.0002**
Supervision	0.10 (0.19)^c^	0.07 (0.14)	0.06 (0.15)^a^	0.06
Worrying	0.56 (0.29)^b,c^	0.32 (0.31)^a^	0.37 (0.31)^a^	**<0.0001**
Urging	0.21 (0.17)^b,c^	0.16 (0.20)^a^	0.21 (0.25)^a^	**<0.0001**
Total score	0.26 (0.15)^b,c^	0.17 (0.16)^a^	0.18 (0.17)^a^	**<0.0001**

Note: *=Consequences-index: the number of “real consequences” divided by the total number of items in the scale. Range 0 (no consequences at all) -1 (maximum level of consequences). *IEQ*: Involment Evaluation Questionnaire. Superscript letters represent statistical differences between groups(^a^=caregivers of patients with eating disorders; ^b^= caregivers of patients with depression; ^c^= caregivers of patients with schizophrenia)

*n*=sample size

*P*-value in bold indicated a significance level of *p*<0.005

The aim of our study was to compare the consequences of being a caregiver for patients with EDs with the consequences of being a caregiver for patients with depression or schizophrenia, all assessed using the same validated instrument, the Involvement Evaluation Questionnaire. We also aimed to identify factors that may predict these consequences. We asked:

- Do caregivers of patients with eating disorders perceive greater caregiving burden than caregivers of patients with depression or schizophrenia?
- What are the predictors of consequences of burden among caregivers of patients with eating disorders, depression, or schizophrenia?
- What is the impact of caring for ED patients?

## Methods

### Study participants

The eating disorders sample consisted of caregivers of patients diagnosed with, and treated for, an ED in the Eating Disorders Outpatient Clinic of the psychiatric services at one hospital and one mental health centre, both in Bizkaia, Spain (ED patients, n = 145; caregivers, n = 251). These institutions, which serve a population of 300,000 inhabitants, are part of the Basque Health Care Service, which provides free, unrestricted care to nearly 100 % of the population. Outpatients diagnosed with anorexia nervosa or bulimia nervosa based on criteria established in the Diagnostic and Statistical Manual of Mental Disorders, 4th edition (DSM-IV) [25] were eligible for the study. Patients were excluded if they had a malignant, severe organic disease, could not complete the questionnaires because of language barriers, or did not give written informed consent to participate in the study. Patients who agreed to participate gave written informed consent. They were asked to name the caregiver who could be asked to participate in the study. The caregiver could be anyone who was involved with the patients, such as a relative, partner or friend, excluding professional caretakers.

Family caregivers were included in the study if they provided written informed consent and the patient for whom they were caring also agreed to participate. Exclusion criteria for the caregivers were the same as for the patients.

The depression sample consisted of caregivers of patients who fulfilled the DSM-IV criteria [25] for major depressive disorder, single episode or recurrent (296.2x, 296.3x), dysthymic disorder (300.40), and other depressive disorders (309.00, 311.00). Patients and caregivers were recruited from three mental hospitals in The Netherlands, all specialized in the treatment of depression (n = 252) [12].

The schizophrenia sample originated from the European EPSILON study [26, 27]. All patients had an ICD-10 [28] diagnosis of schizophrenia. Patients and caregivers were recruited from The Netherlands, Denmark, and The United Kingdom (n = 151) [12].

In both case (depression and schizophrenia) written informed consent was given by patients and caregivers, according to the protocol developed in the Netherlands. Data from both the depression and schizophrenia samples were used with permission of one of the study authors (Dr. van Wijngaarden).

### Measures

Sociodemographic information of the patients was obtained from their medical records. Caregivers provided self-reported sociodemographic data, including age, gender, marital status, level of education, and relationship to the patient. They also completed an instrument to assess their perception of caregiving: the Involvement Evaluation Questionnaire EU Version (IEQ-EU).

The IEQ-EU is a self-reported questionnaire to assess the consequences of being a caregiver. Items are scored on a 5-point Likert scale (0 = never, 1 = sometimes, 2 = regularly, 3 = often, 4 = always), and are grouped into four subscales: (I) tension, such as quarrels or strains in the interpersonal relationship between patient and caregiver; (II) supervision, which covers the caregiver’s duties supervising the patient, such as supervising the intake of medicine or food or preventing suicide; (III) worrying, which is related to the caregiver’s concerns about the patient’s safety, future, and health; and (IV) urging, which evaluates the caregiver’s need to stimulate the patient to undertake activities. Also a 27-item total score can be computed. The IEQ-EU has been translated into and validated in Spanish [8, 29]. It shows good internal consistency and adequate test-retest reliability. The IEQ-EU has been used previously in studies of caregivers of patients with ED, schizophrenia, and depression [8, 11, 30, 31].

The IEQ-EU was sent to the appointed caregiver accompanied by a letter explaining the aims of study, an informed consent form, and a postage-free return envelope. In case of non-response, a reminder was sent after two weeks. The response rate of caregivers was 81 % in the ED sample, 78 % in the depression sample, and 70 % in the schizophrenia sample. The institutional review board of Galdakao-Usansolo Hospital approved this project.

### Data analysis

Descriptive statistics of caregivers’ sociodemographic variables were calculated using means and standard deviations (SD) for quantitative data, and frequencies and percentages for categorical data across the three illness types. Patient mean age and standard deviation were also computed. We evaluated associations between the variables and caregiver group using the Chi-square test (or Fisher’s Exact test when the expected frequencies were lower than 5) for categorical variables, and ANOVA for continuous variables (or Kruskall-Wallis test if normality was rejected).

To study the differences in the IEQ-EU subscale scores between the ED, depression and schizophrenia caregiver samples, so-called “consequences-indexes” (C-index) were computed [12]. IEQ-EU item scores of ≤2 (never, sometimes) were considered as indicating “no consequence”. Item scores of ≥3 (regularly, often or always) were considered as indicating a “real consequence”. For each subscale, the number of “real consequences” was counted and divided by the total number of items in the scale. This led to four subscale C-indexes and one overall C-index, all ranging from “0” (no consequences at all) to “1” (maximum level of consequences). These were our outcome measures. To assess C-index differences among the caregivers of patients with ED, depression, and schizophrenia, we used an analysis of variance (ANOVA) or non-parametric Kruskall-Wallis test if normality was rejected. In addition, we conducted post-hoc tests to identify specific differences between groups, developing a relevant paired test for each pair and using Scheffe test or Wilcoxon test if normality was rejected, and Bonferroni’s correction.

We developed predictive models to determine which variables were relevant to the C-index of each IEQ-EU domain and total score. A univariate analysis was initially performed: Student’s *t* test and ANOVA (or Wilcoxon test and Kruskall-Wallis test if normality was rejected) were used for caregivers’ variables, while a univariate hierarchical linear mixed model was developed for patient age (due to higher number of caregivers than patients). In the multivariate analysis, hierarchical linear mixed models were used for the C-index. In all cases, C-indexes were considered as dependent variables. The intraclass correlation coefficient (ICC) was calculated to assess the correlation among observations within a cluster. All effects were deemed statistically significant at p < .05. All statistical analyses were performed using the SAS System, version 9.2 (SAS Institute, Inc., Carey, NC). The figure was created using the R 2.15 release.

## Results

### Sociodemographic characteristics of patients and their caregivers

Sociodemographic characteristics of the patients and their caregivers are listed in Table 2, grouped by patient diagnosis (ED, depression, schizophrenia). In the analysis of differences between caregivers of patients with ED, depression, and schizophrenia, statistically significant differences were found for all the variables.

**Table 2:** Sociodemographic characteristics of patients with eating disorders, depression, or schizophrenia, and their caregivers

Caregivers of patients with				
	Eating Disorders	Depression	Schizophrenia	p-value
	(n = 251)	(n = 252)	(n = 151)	
***Caregiver variables***	**n (%)**	**n (%)**	**n (%)**	
**Age**				**<.001**
≤45	84 (33.47)	121 (48.21)	39 (25.83)	
45-60	129 (51.39)	86 (34.26)	68 (45.03)	
>60	38 (15.14)	44 (17.53)	44 (29.14)	
**Gender**				**<.001**
Female	135 (53.78)	132 (52.38)	106 (70.20)	
**Relationship of caregiver to patient**				**<.001**
Mother	111 (44.22)	11 (4.37)	65 (43.05)	
Father	70 (27.89)	6 (2.38)	23 (15.23)	
Spouse/partner	34 (13.55)	185 (73.41)	16 (10.60)	
Sibling/child	31 (12.35)	30 (11.90)	27 (17.88)	
Friend	5 (1.99)	20 (7.94)	20 (13.25)	
**Education level**				**<.001**
Primary education	103 (41.87)	31 (12.45)	-	
Secondary education	54 (21.95)	201 (80.72)	-	
Higher education	89 (36.18)	17 (6.83)	-	
**Marital status**				**<.001**
Single	35 (13.94)	25 (9.92)	23 (15.23)	
Spouse/partner	193 (76.89)	211 (83.73)	88 (58.28)	
Divorced	11 (4.38)	12 (4.76)	15 (9.93)	
Widow(er)	12 (4.78)	4 (1.59)	25 (16.56)	
**Contact with patient**				**<.001**
<32 hours/week	137 (55.92)	80 (31.75)	101 (66.89)	
≥32 hours/week	108 (44.08)	172 (68.25)	50 (33.11)	
**Living with the patient**				**<.001**
No	39 (15.66)	32 (13.73)	60 (51.72)	
Yes	210 (84.34)	201 (86.27)	56 (48.28)	
***Patient variables***	**Patients with Eating Disorders** (n = 146)	**Patients with Depression** (n = 252)	**Patients with Schizophrenia** (n = 151)	
**Duration of illness**				**<.001**
≤3 years	53 (36.55)	79 (33.19)	12 (9.09)	
3-10 years	53 (36.55)	77 (32.35)	43 (32.58)	
>10 years	39 (26.90)	82 (34.45)	77 (58.33)	
**Age** ^a^	25.85 (8.94)	45.43 (13.67)	37.83 (11.72)	**<.001**
**Gender**				
Female	144 (98.63)	154 (61.11)	53 (35.10)	**<.001**

*Note*. ^a^mean (standard deviation). n (%) = sample size (percentage), - data not available

P-values in bold indicated a significance level of p < 0.05

The majority of caregivers in the ED sample was mother, lived with her patient, and was married. The mean age of the caregivers was 47.88 (SD = 12.45), and the mean age of the ED patients was 25.58 (SD = 8.96). The majority of caregivers in the schizophrenia sample was the mother of a son with a relatively long history of mental illness, and was married. Almost half of the caregivers in the schizophrenia sample did not live with the patient and spent less time together. The mean age of the caregivers was 53.34 (SD = 13.98), and the mean age of schizophrenic patients was 37.83 (SD = 11.72). The majority of caregivers in the depression sample was the partner of a patient, lived with the patient and spent more time with the patient. The mean age of the caregivers was 46.41 (SD = 13.54), and the mean age of the patients who suffer from depression was 45.43 (13.67). Overall one could say that caregiving in ED generally took place in the household by a mother, while caregiving in depression generally took place in the household by a partner, compared to ongoing parental care for adult children in the schizophrenia sample.

### Comparing caregiving consequences in the eating disorders, depression and schizophrenia samples

**Fig. 1:**
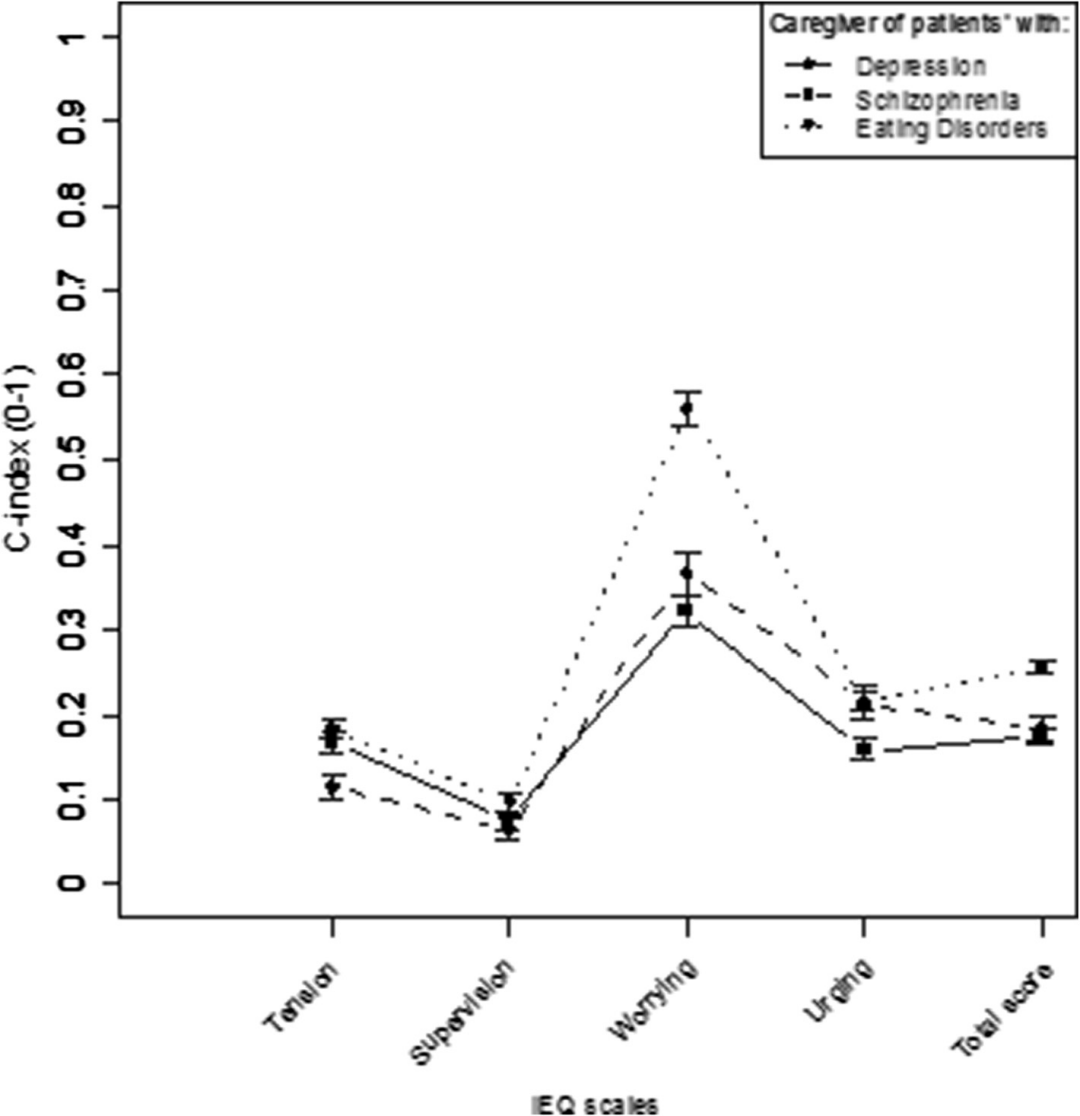
Consequences of caregiving across the three mental illness samples compared: eating disorders, depression, and schizophrenia

The three caregiver samples were compared using the mean IEQ-EU subscale C-index scores (Fig. 1). The samples differed in tension (higher in caregivers of ED and depression), worrying (higher in caregivers of ED), and urging (higher in caregivers of ED and schizophrenia). In all samples, worrying was the most commonly reported consequence. Within each mental illness sample, worrying scale displayed the highest score values whereas the supervision scale showed the highest variation compared to other subscales.

### Univariate analysis of caregiver IEQ-EU scores according to caregiver and patient variables

Caregiver scores on the IEQ-EU are detailed in Table 3 according to univariate analysis of caregiver and patient variables. Higher scores on the tension subscale were associated with a caregiver being a spouse or partner (p < .01) compared to those being friend, spending more than 32 hours per week with the patient (p < .01), living with the patient (p < .001), and younger patient age (p < .05). Higher scores on the supervision subscale were associated with a caregiver age younger than 45 years (p < .05) compared to those caregivers older than 45 years, spending more than 32 hours per week with the patient (p < .01), living with the patient (p < .05), and younger patient age (p < .001). Higher scores on the worrying subscale were associated with being female (p < .05), being the mother of a patient (p < .001) compared to those being spouse or partner, sibling or child or friend, having achieved only primary education (p < .001) compared to those with secondary education, and younger patient age (p < .001). Higher scores on the urging subscale were associated with having achieved only primary education (p < .001) compared to those with secondary education, and living with the patient (p < .01). Higher scores on the total IEQ-EU score were associated with being the mother of a patient (p < .001) compared to those being friend, having achieved only primary education (p < .001) compared to those with secondary education, and living with the patient (p < .001).

**Table 3:** Univariate analysis of the caregivers’ IEQ-EU scores according to caregiver and patient variables

IEQ-EU										
	Tension		Supervision		Worrying		Urging		IEQ-EU Total	
	$\overline{x}$ (SD)	p-value	$\overline{x}$ (SD)	p-value	$\overline{x}$ (SD)	p-value	$\overline{x}$ (SD)	p-value	$\overline{x}$ (SD)	p-value
***Caregiver variables***										
**Age**		0.32		**<.05**		0.29		0.15		0.95
≤45^a^	0.18 (0.21)		0.09 (0.16)^b,c^		0.40 (0.32)		0.19 (0.21)		0.21 (0.17)	
45-60^b^	0.16 (0.19)		0.07 (0.16)^a^		0.44 (0.31)		0.18 (0.20)		0.20 (0.16)	
>60^c^	0.14 (0.18)		0.07 (0.16)^a^		0.44 (0.34)		0.23 (0.23)		0.21 (0.17)	
**Gender**		0.10		0.57		**<.05**		0.29		0.05
Female	0.17 (0.20)		0.08 (0.17)		0.45 (0.32)		0.20 (0.22)		0.22 (0.17)	
Male	0.14 (0.18)		0.08 (0.14)		0.39 (0.31)		0.18 (0.19)		0.19 (0.15)	
**Relationship of caregiver to patient**		**<.01**		0.05		**<.001**		0.20		**<.001**
Mother^a^	0.17 (0.20)^e^		0.09 (0.19)		0.53 (0.30)^c,d,e^		0.21 (0.21)		0.24 (0.17)^e^	
Father^b^	0.14 (0.17)		0.07 (0.14)		0.51 (0.32)^c,e^		0.18 (0.17)		0.21 (0.15)^e^	
Spouse/partner^c^	0.18 (0.20)^e^		0.09 (0.16)		0.34 (0.30)^a,b^		0.20 (0.22)		0.20 (0.17)^e^	
Sibling/child^d^	0.15 (0.20)		0.05 (0.12)		0.41 (0.34)^a^		0.18 (0.21)		0.19 (0.17)	
Friend^e^	0.07 (0.13)^a,c^		0.05 (0.12)		0.26 (0.26)^ab^		0.14 (0.17)		0.12 (0.12)^a,b,c^	
**Educational level**		0.15		0.13		**<.001**		**<.001**		**<.001**
Primary education^a^	0.18 (0.18)		0.10 (0.19)		0.54 (0.32)^b^		0.23 (0.19)^b^		0.25 (0.15)^b^	
Secondary education^b^	0.17 (0.20)		0.07 (0.13)		0.37 (0.31)^a,c^		0.16 (0.19)^a^		0.19 (0.16)^a,c^	
Higher education^c^	0.18 (0.22)		0.11 (0.20)		0.50 (0.32)^b^		0.19 (0.17)		0.24 (0.17)^b^	
**Marital status**		0.58		0.07		0.64		0.51		0.94
Single^a^	0.17 (0.22)		0.09 (0.18)		0.40 (0.32)		0.20 (0.22)		0.21 (0.18)	
Married/partner^b^	0.16 (0.19)		0.08 (0.15)		0.42 (0.32)		0.19 (0.20)		0.21 (0.16)	
Divorced^c^	0.19 (0.24)		0.15 (0.23)		0.49 (0.34)		0.16 (0.21)		0.23 (0.21)	
Widow(er)^d^	0.12 (0.16)		0.05 (0.17)		0.43 (0.32)		0.20 (0.21)		0.19 (0.16)	
**Contact with patient**		**<.01**		**<.05**		0.15		0.14		0.37
≤32 hours/week	0.14 (0.19)		0.07 (0.14)		0.44 (0.31)		0.18 (0.19)		0.20 (0.16)	
≥32 hours/week	0.18 (0.20)		0.09 (0.17)		0.41 (0.33)		0.21 (0.22)		0.22 (0.17)	
**Living with the patient**		**<.001**		**<.001**		0.13		**<.01**		**<.001**
No	0.08 (0.13)		0.04 (0.12)		0.39 (0.34)		0.15 (0.18)		0.16 (0.14)	
Yes	0.19 (0.20)		0.09 (0.17)		0.44 (0.31)		0.21 (0.21)		0.23 (0.17)	
***Patient variables***										
**Duration of illness**		**<.05**		0.33		0.48		0.20		0.13
≤3 years^a^	0.19 (0.20)^b^		0.10 (0.18)		0.42 (0.29)		0.22 (0.22)		0.23 (0.17)	
3-10 years^b^	0.15 (0.20)^a^		0.07 (0.15)		0.42 (0.33)		0.18 (0.20)		0.20 (0.17)	
>10 years^c^	0.15 (0.19)		0.08 (0.16)		0.39 (0.33)		0.20 (0.23)		0.20 (0.17)	
**Age** ^**§**^	-0.001 (0.0005)	**<.05**	-0.0008 (0.0005)	0.07	-0.005 (0.0009)	**<.001**	0.0005 (0.0006)	0.39	-0.001 (0.0005)	**<.01**
**Gender**		0.73		0.69		0.29		0.85		0.48
Female	0.16 (0.19)		0.08 (0.17)		0.41 (0.32)		0.19 (0.20)		0.20 (0.16)	
Male	0.16 (0.20)		0.08 (0.15)		0.38 (0.32)		0.20 (0.23)		0.20 (0.18)	

*Note.* $\overline{x}$ (*SD*) mean (standard deviation), ^§^Beta (standard error), *IEQ* Involvement Evaluation Questionnaire, Superscript letters in the first column represent statistical differences between groups

P-values in bold indicated a significance level of p < 0.05

### Multivariate analysis of caregivers’ IEQ-EU subscales

Results of the multivariate analysis for the IEQ-EU subscales are presented in Table 4. In the tension subscale, being the caregiver of an ED patient (p < .05) compared to those being the caregiver of a patient with schizophrenia, being a patient’s mother or partner (p < .05and p < .01 respectively) compared to those being friend, or younger patient age (p < .05) were predictive for a greater caregiver burden. In the supervision subscale, being the caregiver of an ED patient (p < .05) compared to those being the caregiver of a patient with depression, being a patient’s partner (p < .05) compared to those being friend, or being divorced (p < .01) compared to those being widow(er) were predictive for a greater caregiver burden. In the worrying subscale, being the caregiver of an ED patient p < .001) compared to the other patients’ group, or being a patient’s mother or father (p < .001and p < .01, respectively) compared to those being friend were predictive for a greater caregiver burden. In the urging subscale, being the caregiver of an ED patient (p < .001) compared to those being the caregiver of a patient with schizophrenia or being a patient’s partner (p < .01) compared to those being friend were predictive for greater caregiver burden.

**Table 4:** Multivariate analysis of caregivers’ IEQ-EU subscales

			IEQ-EU subscales					
	Tension		Supervision		Worrying		Urging	
	Beta (s.e.)	p-value	Beta (s.e.)	p-value	Beta (s.e.)	p-value	Beta (s.e.)	p-value
**Intercept**	0.16 (0.04)	**<.001**	0.05 (0.04)	0.20	0.42 (0.05)	**<.001**	0.23 (0.04)	**<.001**
**Caregivers of patients with**								
Depression (n = 252)	-0.01 (0.02)	0.62	-0.05 (0.02)	**<.05**	-0.18 (0.04)	**<.001**	-0.11 (0.03)	**<.001**
Schizophrenia (n = 151)	-0.05 (0.02)	**<.05**	-0.04 (0.02)	0.05	-0.19 (0.03)	**<.001**	-0.01 (0.02)	0.75
Eating Disorders (n = 251)	Reference		Reference		Reference		Reference	
**Relationship of caregiver to patients**								
Mother	0.07 (0.03)	**<.05**	0.04 (0.03)	0.12	0.19 (0.05)	**<.001**	0.03 (0.04)	0.44
Father	0.02 (0.04)	0.54	0.02 (0.03)	0.51	0.16 (0.05)	**<.01**	-0.01 (0.04)	0.74
Spouse/partner	0.10 (0.03)	**<.01**	0.07 (0.03)	**<.05**	0.09 (0.05)	0.09	0.09 (0.03)	**<.01**
Sibling/child	0.06 (0.03)	0.08	-0.01 (0.03)	0.63	0.11 (0.05)	0.04	0.02 (0.04)	0.53
Friend	Reference		Reference		Reference		Reference	
**Marital status of caregiver**								
Single	-		0.05 (0.03)	0.11	-		-	
Married/partner	-		0.01 (0.03)	0.69	-		-	
Divorced	-		0.10 (0.04)	**<.01**	-		-	
Widow(er)	-		Reference		-		-	
**Caregiver’s age**								
≤45	-		-		-		-0.05 (0.02)	0.06
45-60	-		-		-		-0.05 (0.02)	**<.05**
>60	-		-		-		Reference	
**Patient age**	-0.002 (0.0007)	**<.05**	-		-		-	
**ICC**	0.34		0.38		0.44		0.61	

Note. (*s.e*.) standard error, *IEQ* Involvement Evaluation Questionnaire, - variable not considered in the final model. Reference: Reference category group. *ICC* Intraclass Correlation Coefficient. A positive estimate indicates an increment in caregiver burden. *IEQ* Involvement Evaluation Questionnaire

P-values in bold indicated a significance level of p < 0.05

### Multivariate analysis of caregivers’ IEQ-EU total score

Results of the multivariate analysis performed for the total IEQ-EU score are presented in Table 5. Predictive variables for a high level of caregiver burden included being the caregiver of an ED patient (p < .001) compared to the other patients’ group and being a patient’s mother or partner compared to those being friend (p < .01).

**Table 5:** Multivariate analysis of caregivers’ total IEQ-EU score

	IEQ-EU total score	
	Beta (s.e.)	*p*-value
Intercept	0.20 (0.03)	**<.001**
Caregivers of patients with		
Depression (n = 252)	-0.09 (0.02)	**<.001**
Schizophrenia (n = 151)	-0.07 (0.02)	**<.001**
Eating Disorders (n = 251)	Reference	
Relationship of caregiver to patients		
Mother	0.07 (0.03)	**<.01**
Father	0.04 (0.03)	0.19
Spouse/partner	0.08 (0.03)	**<.01**
Sibling/child	0.05 (0.03)	0.10
Friend	Reference	
**ICC**	0.47	

Note. (*s.e*.) standard error, *IEQ* Involvement Evaluation Questionnaire, Reference: Reference category group. A positive estimate indicates an increment in caregiver burden. *IEQ* Involvement Evaluation Questionnaire, *ICC* Intraclass Correlation Coefficient

*P*-values in bold indicated a significance level of p < 0.05

## Discussion

This study had two aims, 1) to assess the consequences for caregivers of ED patients, and to compare the consequences for caregivers of ED patients with those of caregivers of patients with depression and schizophrenia; and 2) to identify factors that may predict these consequences for caregivers of all three samples. A substantial finding of our study is that the caring consequences of caregivers of ED are higher than the consequences for caregivers who provide care for patients with schizophrenia and depression.

In our study, the caregiving consequences are very similar regarding the patterns of consequences within the three samples. In all samples worrying was reported most often and supervision least often. Worrying covers painful interpersonal cognitions, such as concern about the patient’s safety, general health, and the kind of help he or she is receiving, while supervision has to do with the caregiver’s tasks of ensuring and guarding, for instance, the patient’s intake of medicine, sleep, and dangerous behavior [19].

We identified different predictors of caregiver burden in the three samples of patients (ED, depression and schizophrenia). In terms of patient variables, patient age was associated with tension: the younger the patient, the higher the caregiver burden, probably because the caregivers feel that they do not have the control over the situation, and most of the time they do not know how to cope with that situation [32]. Among younger ED patients the parents’ loss of control over their son’s or daughter’s eating may result in their feeling that they have failed to fulfil a basic parenting task [33]. Role performance could explain this since relatives of older patients may be more resigned to the effects of illness and more accepting of their caregiving [34].

Caregiver variables associated with higher caregiver burden included the type of illness as well as the caregiver’s relationship with the patient, marital status, and age. Caregivers of patients with ED showed higher perceived burden than caregivers of patients with schizophrenia. This burden most likely resulted from tension in the relationship that arises from giving care; higher worry, related to worrying about the patient’s health and future, and higher total burden. Compared to caregivers of patients with depression, caregivers of patients with ED reported more supervision of their patients; a greater need to urge the patient to do things; more worrying, related to worrying about the patient’s health and future; and more total burden. Caregivers of ED patients must often supervise their patients’ eating behaviours (encouraging them to eat more or less), persuade them to change compulsive behaviours, help prevent social isolation, withstand mood swings, and worry about their future [35]. The patient’s behavioural changes may disrupt the family life; family daily routines are usually disrupted, with mealtime becoming a battle that causes distress for the entire family. The family eating pattern is often altered in an attempt to help the patient eat. Carers may end up preparing different foods at different times for different family members and meals are no longer a social event, but a struggle that adds to the carer’s distress. [36].

The type of caregiver (i.e., the relation of caregiver to patient: mother, father, spouse or partner, sibling or child, friend) was an important predictor of caregiving consequences. Caregivers of patients with ED, depression or schizophrenia who were partners or mothers had higher caregiver burden. Compared to friends who were caregivers, partners who were caregivers had higher tension, supervision, urging, and overall burden; mothers had higher tension, worrying, and overall burden; and fathers had higher worrying. Mothers may perceive a higher caregiving burden than fathers because mothers may be in closer contact with their child, feel more responsible for the disorder, or are more affected by their child’s relapses or crises [24]. Mothers show the highest level of burden, probably because they usually are responsible for the main part of the patient’s care [32]. Kung [37] relates this fact to a higher level of involvement needing even a more active approximation from the professionals of mental health [38]. It is possible that mothers and partners are most affected by providing care to a patient with an ED due to the fact that they often undertake supervisory roles at mealtimes [30]. It is also possible that mothers adopt a more emotional coping style than fathers, which may lead to greater stress [39]. The lower impact on fathers could also be due to their lower involvement in practical care roles, or because their coping style is aimed more towards problem-solving [40]. Depression tends to manifest itself in adulthood. The person one has married and with whom one is engaged in an emotional and sexual relationship has changed, and future life suddenly looks quite different [12]. In schizophrenia are mainly mothers who assume tasks otherwise performed by the patient himself or herself [1], considering that first episodes generally occur in adolescence. Most of the studies on burden show that the mother who takes care of the patient with schizophrenia shows the highest burden even if they share the task with other relatives [41–44]. They were typically responsible for most aspects of the patient’s daily care, such as overseeing pharmacological treatment, ensuring that the environment is calm, controlling alcohol or other drug use, helping patients to manage their free time and dealing with everyday difficulties, all of which constitutes a significant source of stress.

Being divorced appears to have consequences for caregivers. In our study, divorced caregivers reported greater perceived care burden resulting from routine supervision of the patients (as the widow/er). Divorce results in disruption of the marital relationship and family life, and may lead to more limited social support for the caregiver [16]. A divorced caregiver will not have a spouse to share the burden of caregiving with. In addition, there are probably more financial stresses, if the caregiver is both earning a living and caregiving. The caregiver might also have other dependents to look after too – such as other children, or parents who need support [45].

Higher caregiver age (>60 years) was associated with a higher burden resulted from the need to urge the patient to do things, and so, higher consequences of burden on urging. This result is consistent with the findings of other studies [46], where older caregivers show higher burden because they have coexisted for longer with the patient and the disorder. Possible explanations may be, for example, that the youngest caregivers have a more stressful family and social life situation than the older caregivers, the families become smaller, and the changes in health and medical care, have resulted in the family members becoming care providers [9].

### Strengths and limitations

Our study has several strengths. These include the use of a validated instrument to determine caregiver burden; a large sample of caregivers; and patients with three types of mental illnesses.

Several limitations must also be noted. Our study included only family caregivers of outpatients. Thus, the results will not necessarily generalize to other settings, such as inpatients or patients treated as part of primary care. Another limitation concerns the differences between the three samples, a result of the contexts in which caregiving takes place, and possible cultural differences. Van Wijngaarden et al. (2000) found important cultural differences in the extent and nature of caregiving between European countries (8). In our study, caregiving in eating disorders, caregiving in depression and caregiving in schizophrenia inevitably take place in a different context, with different role shifts, both in patient and caregiver. Nevertheless, one finding of this study is that the caregiving consequences are very similar on the patterns of consequences within the samples. In the three samples worrying was reported most often and supervision least often. Another limitation was that we did not examine additional factors that may account for variations in caregivers’ consequences, such as the severity of symptoms in all patient samples. That was because we did not have a common instrument to assess severity of symptoms across the three disorders evaluated: EDs, depression, and schizophrenia. This was inevitable due to the study design, which involved in part secondary data analysis. An additional limitation was that we assessed caregivers and patients at only one point in time, which did not allow us to observe changes in caregiver burden over time or make any statements about causation.

## Conclusions

The findings of this study confirm that the consequences of providing care to ED patients are higher than those perceived by caregivers of patients with depression or schizophrenia. Our results reinforce the hypothesis that caring for a patient with an ED can impose a substantial burden. These findings have clinical implications, highlighting the importance of supporting the caregiver and empowering him or her with skills to tackle the consequences of caregiving.

## Abbreviations

ED: Eating disorders
IEQ: Involvement Evaluation Questionnaire
DSM-IV: Diagnostic and Statistical Manual of Mental Disorders, 4th edition
SD: Standard deviations
C-index: Consequences-indexes
ANOVA: Analysis of variance
ICC: Intraclass correlation coefficient
